# Biological Properties of Low-Toxicity PLGA and PLGA/PHB Fibrous Nanocomposite Implants for Osseous Tissue Regeneration. Part I: Evaluation of Potential Biotoxicity

**DOI:** 10.3390/molecules22122092

**Published:** 2017-11-29

**Authors:** Izabella Krucińska, Bogusława Żywicka, Agnieszka Komisarczyk, Maria Szymonowicz, Stanisława Kowalska, Ewa Zaczyńska, Marcin Struszczyk, Anna Czarny, Piotr Jadczyk, Barbara Umińska-Wasiluk, Zbigniew Rybak, Marek Kowalczuk

**Affiliations:** 1 Department of Material and Commodity Sciences and Textile Metrology, Technical University of Lodz, Zeromskiego 116, 90-924 Lodz, Poland; izabella.krucinska@p.lodz.pl (I.K.); skowal@p.lodz.pl (S.K.); marcin.struszczyk@hotmail.com (M.S.); 2 Department of Experimental Surgery and Biomaterials Research, Wroclaw Medical University, Pasteura 1, 50-367 Wroclaw, Poland; boguslawa.zywicka@umed.wroc.pl (B.Ż.); maria.szymonowicz@umed.wroc.pl (M.S.); zbigniew.rybak@umed.wroc.pl (Z.R.); 3 Institute of Immunology and Experimental Therapy, Polish Academy of Sciences, RudollfaWeigla 12, 53-114 Wroclaw, Poland; ezacz@iitd.pan.wroc.pl (E.Z.); czarny@iitd.pan.wroc.pl (A.C.); 4 Department of Sanitary Biology and Ecotechnics, Wroclaw University of Technology, Wybrzeze Wyspianskiego 27, 50-370 Wroclaw, Poland; piotr.jadczyk@pwr.edu.pl (P.J.); barbara.uminska-wasiluk@pwr.edu.pl (B.U.-W.); 5 Centre of Polymer and Carbon Materials of the Polish Academy of Sciences, MariiSkłodowskiej-Curie 34, 41-819 Zabrze, Poland; marek.kowalczuk@cmpw-pan.edu.pl or M.Kowalczuk@wlv.ac.uk; 6 Faculty of Science and Engineering, University of Wolverhampton, Wulfruna Street, WV1 1SB Wolverhampton, UK

**Keywords:** biotoxicity, biodegradation, cytotoxicity, genotoxicity, hemocompatability, nonwoven fabrics, bone implant, poly(l-lactide-*co*-glycolide), synthetic poly([*R,S*]-3-hydroxybutyrate), encapsulated growth factor

## Abstract

In response to the demand for new implant materials characterized by high biocompatibility and bioresorption, two prototypes of fibrous nanocomposite implants for osseous tissue regeneration made of a newly developed blend of poly(l-lactide-*co*-glycolide) (PLGA) and syntheticpoly([*R,S*]-3-hydroxybutyrate), PLGA/PHB, have been developed and fabricated. Afibre-forming copolymer of glycolide and l-lactide (PLGA) was obtained by a unique method of synthesis carried out in blocksusing Zr(AcAc)_4_ as an initiator. The prototypes of the implants are composed of three layers of PLGA or PLGA/PHB, nonwoven fabrics with a pore structure designed to provide the best conditions for the cell proliferation. The bioactivity of the proposed implants has been imparted by introducing a hydroxyapatite material and IGF1, a growth factor. The developed prototypes of implants have been subjected to a set of in vitro and in vivobiocompatibility tests: in vitro cytotoxic effect, in vitro genotoxicity and systemic toxicity. Rabbitsshowed no signs of negative reactionafter implantation of the experimental implant prototypes.

## 1. Introduction

Medical textiles play an important role in alloplastic implantology. The fibrous structure of textiles and broad possibilities for surface and structure modifications make them irreplaceable in the reconstruction of natural tissues of the human body. In the process of designing implantable medical textile products serving as scaffolds for tissues, the set of naturally occurring properties of each product should be recognized.

The expected substrate for cell growth should be biocompatible, but also bioactive to ensure the proliferative ability of the settled cells. In the next regeneration stages, the substrate should become a support base for the expanding tissue and in this respect should be characterized by the compatibility of its structural and mechanical properties with the newly formed tissue. After the formation of regenerated tissue, avoiding another surgery will be possible if the substrate undergoes resorption without producing toxic molecules. The dynamics of resorption should be correlated with the dynamics of restoring a given type of tissue in the organism. Currently, among resorbable materials, polymer materials are of great importance. These materials can be divided into natural and synthetic categories. Among the natural materials, most research is carried out on polypeptides in the form of collagen and fibroin as well as polysaccharides, i.e., chitin derivatives, hyaluronic acid or alginates [1,2,3,4,5,6]. Among the synthetic polymers, aliphatic polyester polymers are of great interest. The main representatives of this group are poly(lactide) (PLA), poly(glycolide) (PGA), polycaprolactone (PCL) and their copolymers as well as polyhydroxybutyrates (PHB) [7,8,9,10,11,12]. Considering the susceptibility of the polymer to bioresorption, the time of disintegration of polylactide depends on the construction of the polymer chain. Atactic poly(LLA-co-DLA), built from heterochiral l,d-PLA chains and having a random distribution of the l- and D-PLA forms, is amorphous. This type of supramolecular structure contributes to faster polymer bioresorption. Therefore, for cellular substrates preparation, the most commonly used is a copolymer of both of the two optically active isomeric forms of lactide. Lactic acid is a degradation product that is normally present in the human body and is excreted in metabolic processes. Another way to modify the resorption time is to insert a glycolide repeat unit in the structure of the polymer [7,9,10]. Poly(lactide-*co*-glycolide) (PLGA) is a copolymer that is obtained in this way. Tin compounds are predominantly used for the synthesis of PLGA [11,12]. It has been shown, however, that organic and inorganic tin compounds, especially when administered by parenteral routes, exhibit different degrees of toxicity. Increased supply of tin causes accumulation in the liver and kidneys, but also in the lungs and bones. Some tin salts can cause renal necrosis after parenteral doses. The main results of tin toxicity are skin and eye irritation; cholangitis of the lower biliary tract, and later hepatotoxicity; and neurotoxicity. The WHO has outlined the admissible doses of tin and its compounds [13]. The PLGA synthesis methods have also been developed using zinc, calcium and zirconium compounds [14,15,16]. The non-toxic in vitro PLGA polymer, synthesized using zirconium acetylacetonate and patented by the Centre of Polymer and Carbon Materials Sciences in Zabrze [17], was the base for the new implants for osseous tissue regeneration investigated in this article [18]. The structure of the materials designed as an implant or scaffold plays also important role in the process of healing of the tissue. Such materials are a solid framework for cell growth and differentiation at a local site, allowing cell attachment and migration when applied to bone tissue regeneration enable osteoconductivity. Depending on the situation, the scaffold may be implanted alone to induce host cell migration to the wound site and initiate tissue regeneration, or it may serve as a carrier for cells [19]. The properties of tissue scaffolds determine the development of engineered tissues because the 3-D scaffold is in direct contact with cells and it provides physical cues to regulate cell proliferation and differentiation. It is known, that both porosity and pore size have direct influence on their functionalities for biomedical applications [20,21,22]. The best ingrowing of the cells goes when the porosity of the material is high, over 80% and the pore size diameter is higher than 100 μm up to 750 μm [2]. Other side, such porosity can cause, that the cells are able to move out from the structure and growing outside of the material, especially, when the scaffold is used as a carrier of the cells. Moreover, studies found that cell growth and function were closely related to the surface geometry of substrate by using micropatterned or microgrooved surfaces [23]. Thus, the design of the construction of the material incorporated to the living organism should include not only the macroporous part but also microporous surface, suitable for cell colonisation and limiting cell migration. Such constructions may be supplemented with innovative targeted therapeutic substances that increase bioactivity, e.g., nanoparticles containing HAp for bone tissue remineralisation. There are also possibilities of the application of biochemical signals, in the form of growth factors, cytokines, genes, or other environmental cues [19]. The difference in surface chemistry could be sensed by certain types of cells. Cell proliferation and differentiation can be regulated through extracellular matrix (ECM) proteins adhering on surface. If the surface was directly coated using ECM protein solution (e.g., fibronectin or laminin) with different concentrations, cell shape can be controlled by adjusting ECM density [24]. Some research incorporated various growth factors, like photoreactive epidermal growth factor (EGF) which was immobilized on a micropatterned surface [25]. There are number of the grow factors, like fibroblast growth factor-2 (FGF-2) or heparin-binding epidermal growth factor-like growth factor, which can be applicable during scaffold design, dependently on the type of the cells and tissue which can be used [26,27,28,29]. Application of the grow factors increases the proliferation of the cells, influences on their orientation, differentiation and alignment. Growth factors such as bone morphogenetic proteins (BMPs), fibroblast growth factor (FGFs), vascular endothelial growth factor (VEGFs) and insulin-like growth factors 1 (IGF1) have significant impacts on osteoblast behavior, and bone tissue regeneration [29]. Recently the important role of IGF1 is being extensively studied. Demonstrated participation in angiogenesis, osteoblast and osteoclast differentiation, modification of BMP activity in such a way that it enhances its effect, show that IGF1 is a promising supplement for innovative intraosseous fiber implants [30,31,32]. Securing biological stability for a sustainable and controllable release to the target tissue is a challenge to practical applications. This challenge has been addressed to some degree with the development of appropriate carrier materials and delivery systems [29]. One of them is the enclosure of active agents in microspheres. Biological safety and potential toxicity of complex medical devices, especially those containing nanostructures, is a subject of extensive research [33]. Some non-toxic substances in vitro after exposure to metabolic fractions of the living organism may exhibit genotoxicity or mutagenicity [34].

The aim of the study was to design two prototypes of fibrous, bioactive, innovative implants for osseous regeneration using the copolyester PLGA or its blend with poly([*R,S*]-3-hydroxybutyrate) PLGA/PHB with an assessment of their potential toxicity. The innovation of developed implants results from application of polymer with more biocompatible properties, developed porous structure and incorporation to the structure of the material of bioactive compounds (IGF1) in the form of microspheres which enables prolonged time of delivery to the tissue.

The syntheses of these copolymers were developed with the use of Zr(AcAc)_4_. The designed fibrous implants are characterised by their micro- and macroporous structure, which creates an optimal environment for cell migration and proliferation. Additional functionalisation of the implants is obtained by incorporation of insulin-like growth factor (IGF1) and HAp nanoparticles. The safety of the developed biodegradable, nanocomposite non-woven fabrics were verified with a series of biocompatibility tests in vitro such as cytotoxicity and genotoxicity and in vivo such as chronic toxicity after implantation [35]. Assessment of the local effects after implantation and activity of the developed material will be presented in subsequent publications.

## 2. Materials

### 2.1. Structure of the Designed Bone Implant

**Figure 1:**
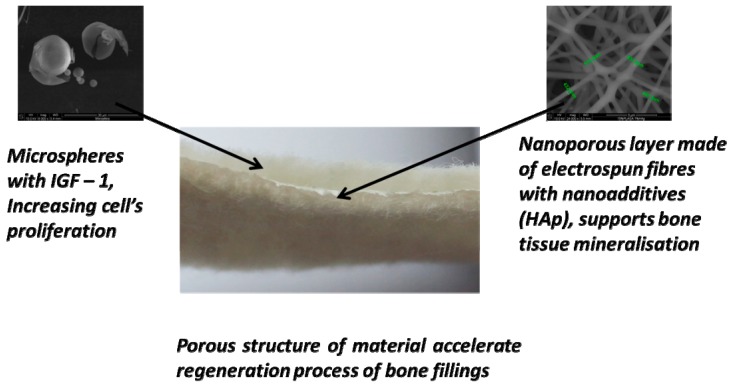
Structure of the implants for osseous tissue regeneration.

The bone implant prototypes were designed as multi-layered structures with the addition of the bioactive elements. The scheme of the designed prototypes is shown in Figure 1. The outer layers of the proposed implant should allow for the penetration of tissue culture inside the scaffolds. Therefore, they were made from needle-punching non-woven fabric characterized by a macroporous structure. The bioactivity of the proposed device is imparted by introducing a hydroxyapatite material and a biologically active agent, an insulin-like growth factor (IGF1, Sigma Aldrich, St. Louis, MO, USA). The role of the middle layer is to immobilize the microspheres with IGF1 and also the penetrating cells. The middle nanoporous layer is composed of electrospun fibres reinforced with ceramic hydroxyapatite nanoparticles. One of the designed prototypes was based on PLGA, and the other was based on the polymeric blend PLGA/PHB. 

### 2.2. Polymers Characteristic

To ensure the high biocompatibility and resorbability of the two designed prototypes of fibrous nanocomposite implants for osseous tissue regeneration, fibre-forming copolymers of glycolide and l-lactide (PLGA) were used at the optimal composition for fibre formation, approximately 15 mol % glycolide and 85 mol% l-lactide, which was obtained by a unique method of synthesis carried out in blocks using Zr(AcAc)_4_ as a initiator, according to the modified method described in [15,16]. Due to the mechanism of the copolymerization initiation, there were no other co-initiators or catalysts used other than the zirconium complex. The PLGA copolymer created using this method clearly has a segmented chain microstructure. The final copolymer chain contains both short segments resulting from the intermolecular transesterification and the glycolide, lactide and lactate units but also the long lactide chain sequences [36]. The average molecular weight determined by GPC was 200,000 g/mol, and the polydispersity index was 1.54. A thermal study using a Differential Scanning Calorimetry (DSC) method showed that the polymer has an amorphous structure and the glass transition temperature is 54 °C. The second material that was examined was a blend of the two polymers that contains the above-described PLGA copolymer and an amorphous poly([*R,S*]-3-hydroxybutyrate) (PHB) in a 9:1 ratio. PHB obtained by anionic polymerization of racemic β-butyrolactone initiated with tetrabutylammonium acetate and finished with hydrochloric acid to obtain the carboxyl terminal groups [37,38]. The synthesis process was carried out in the range of 20 –30°C to attain a monomer conversion above 85%. The resulting PHB was characterized using gel permeation chromatography (GPC) and nuclear magnetic resonance (^1^H- and ^13^C-NMR). As determined by GPC, the weight average molecular weight of PHB was 230,000 g/mol and the dispersity index was 2.3. DSC studies showed that it is an amorphous polymer. Based on the measurements performed by the NMR technique, the obtained polymer was found to be fully atactic. The triads determined from the ^13^C-NMR spectra (using carbon signals from the methylene group) amounted to I = 24%, S = 25%, Hs = 25%, and Hi = 26%. The amount of isotactic dyads (i) calculated from the ^1^H-NMR spectra (signal corresponding to the protons of the groups CH_3_ at 1.28 ppm) was equal to 50% [36].

### 2.3. Fibres Characteristic

The macroporous outer layers of developed prototypes of bone implants were manufactured using fibres delivered by Institute of Biopolymers and Chemical Fibres, the characteristics of which are given in Table 1.

### 2.4. Characteristic of the Macroporous Layer

Because the main objective of the work was the modelling of the porous structures, the study also included the determination of pore size distribution, mean pore size and total pore area. These studies were carried out by Hg porosimetry, which comprises introducing mercury in the porous structure of the test material. With the increase of pressure, the injection of mercury is introduced into pores of diminishing size. The results are shown as the cumulative pore area as a function of their diameter and intrusion versus pore sizes and are in tabular form. The study was carried out in the pressure range 0.001–414 MPa, corresponding to a pore diameter between 400,000 nm and 3.5 nm. The characteristics of needle-punching nonwoven fabrics used as outer layers of both prototypes of implants for osseous tissue regeneration are given in Table 2.

A layer with a pore size of several hundred microns was sandwiched with a layer of nanoporous membrane. The nanoporous membrane was produced by the electrospinning method. Nanofibres were formed from PLGA and a mixture of PLGA/PHB on a 32-point stand for electrospinning. The nanofibres were spun from 15% solutions of PLGA and PLGA/PHB in DMSO with a 1% addition of HAp. The supply voltage was 33 kV, the distance between the head and the collector was 25 cm. In the case of forming fibres from PLGA solutions, the obtained fibres had an average size of 0.47 µm, whereas in the case of forming fibres from PLGA/PHB solutions, the obtained fibres had an average size of 0.18 µm. The selection of the optimal forming conditions of the discussed nanofibers was conducted according to the data presented in paper [39], properties of non-woven fabric layers constituting the implant, are shown in Table 3.

Next, the three-layer material was build using the needle-punching process. Three-layer materials produced from PLGA and PLGA/PHB were used as the substrate for immobilization of the insulin-like grow factor IGF1 encapsulated in calcium alginate microspheres. The microspheres were formed from a 5 wt. % solution of sodium alginate in biologically pure water, to which was added 25 µg IGF1 per 100 mL polymer solution. Microspheres were formed on a laboratory stand for that purpose and solidified in a 10 wt. % solution of calcium carbonate Ca_2_Cl·6H_2_O in biologically pure water. During the solidification, the replacement of the Na^+^ by Ca^2+^ ions in the alginate salt was observed. Decanted microspheres were filtered and then suspended in the biologically pure water. The suspension of microspheres was filtered through the three-layer material. The characteristics of three-layer nonwovens are given in Table 4 and Table 5. 

The total pore area decreases without important changes in pore diameter, which can be connected with filling of the pores by microspheres. Moreover, that incorporation of the microspheres with IGF1 does not cause important changes of mechanical properties of the material. Slightly increasing the recovery after compression for samples containing IGF1 in comparison to samples without IGF1 is connected with the location of the microspheres, mostly at the surface of the material. All variants were sterilized using a radiation dose of 28 kGy (before the IGF1 introduction) and a dose of 15 kGy after IGF1 introduction into the fibrous implant, in validated condition according to PN-EN ISO 11137-2 [40]. Sterilisation does not influence the structural parameters of the products.

## 3. Methodology

### 3.1. Assessment of Technical Properties

The number average molar mass (Mn) of the polymers was assessed by GPC experiments conducted in chloroform solution at 35 °C and a flow rate of 1 mL/min using a 8800 solvent delivery system (Spectra-Physics, Santa Clara, CA, USA) equipped with two Mixed C Styragel columns (Waters Corporation, Milford, MA, USA) in series and a SE 61 refractive index detector (Shodex, Munich, Germany). A volume of 10 μL of sample solutions in CHCl_3_ (concentration 0.5% *w*/*v*) were injected into the system. Polystyrene standards with low polydispersity were used to generate a calibration curve. Changes in the chain microstructure as well as in the copolymer composition were monitored on the basis of ^1^H-NMR and ^13^C-NMR spectra. The ^1^H-NMR spectrum of the PLGA were recorded at 600 MHz andambient temperaturewith anAvance II Ultrashield Plus spectrometer (Bruker Polska, Poznan, Poland) and a 5 mm sample tube. Dried deuterated chloroform was used as solvent and tetramethylsilane was used as the internal standard. All ^1^H-NMR spectra were acquired with 32 scans, a 2.65 s acquisition time and an 11 µs pulse width. The ^13^C-NMR spectra of the PLGA were recorded at 150 MHz with the same spectrometer and conditions as the proton spectra. The acquisition time was 0.9 s, the pulse width was 9.4 µs, the delay between pulses was 2 s, and the spectral width was 36,000 Hz. The copolymer composition was calculated based on integration of protons of methylene region of glycolidyl units (at δ = 4.6–4.9 ppm) and methine region of lactidyl units (at δ = 5.1–5.3). The average length of the lactidyl and glycolidyl microblocks was calculated by measuring the signal intensities of the carbonyl carbons, according to the method described by us earlier [14]. Differential scanning calorimetry (DSC) was run on a Pyris Diamond DSC (Perkin Elmer, Waltham, MA, USA) at 10 °C/min, under nitrogen atmosphere. Characteristic of the fibres was assessed according to European standards. Linear mass was assessed according to EN-ISO 1973:2011 [41] standard, point 4.1. Mechanical properties of fibres, including tenacity and elongation was done using Instron model 5944Tensile Tester Machine (Instron, Norwood, MA, USA), according to the EN ISO 5079:1999 [42] standard. Shrinkage in water at 60 °C was done according to PN-87/P-04761.05 [43] standard. The degree of crystallinity of the polymer was tested using a wide-angle X-ray diffractometer (WAXS) using the X’Pert Pro System (PANalytical, Almelo, The Netherlands). The diffraction patterns were obtained using Cu Kα rays (λ = 0.154 nm), with the following lamp performance parameters: acceleration voltage—40 kV and anode current pressure—30 mA. Samples were tested in powder form. The degree of crystallinity was evaluated using WAXSFIT software [44]. Diffractograms were analysed using the Hindele and Johnson methods. The size of the crystals was analysed using measurement of, the width of the diffraction peak by the Scherrer formula. The apparent density was calculated according to the following formula:

δ = M_p_/d, (kg/m^3^)

where Mp is surface mass in g/m^2^, and d means the thickness of material in mm.

Each layer of the implant as well as the final product was investigated by basic physical parameters: surface mass, thickness, apparent density and the porous structure. The surface mass was tested in accordance with EN 29073-1 [45], and the thickness was tested in accordance with EN ISO 9073-2 [46]. The air permeability was tested using a FX 3300-III apparatus (Textest Instruments, Schwerzenbach, Switzerland), according to the EN ISO 9237:1998 [47]. With the difference of the pressure 100 Pa and measuring area 20 cm. All tests of fibres and non-wovens were done under standard controlled climatic conditions (20 °C and 65% RH) [48]. Because the main objective of the work was the modelling of the porous structures, the study also included the determination of pore size distribution, mean pore size and total pore area. These studies were carried out by Hg porosimetry, which involves introducing mercury in the porous structure of the test material. With the increase of pressure, the injection of mercury is introduced into pores of diminishing size. The results are shown as the cumulative pore area as a function of their diameter and intrusion versus pore sizes and are in tabular form. The study was carried out in the pressure range 0.001–414 MPa, corresponding to a pore diameter between 400,000 nm and 3.5 nm [49].

### 3.2. Biological Studies

The investigations of the in vitro cytotoxicity and its influence on cell culture were conducted with the use of all manufactured types of implants containing the insulin-like growth factor IGF1 i.e., PLGA + IGF1 and PLGA/PHB + IGF1, as well as the use of the control samples of the developed implants without a growth factor, PLGA, and PLGA/PHB. Next the evaluated implants PLGA + IGF1 and PLGA/PHB + IGF1 were subjected to the evaluation of their genotoxicity in the Ames test and also the chronic toxicity using an animal model. The I Local Ethics Committee for Animal Experimentation in Wroclaw accepted the proposed and currently shown animal studies as admissible (consent no. 11/2008 and 88/2012, 89/2012 and 90/2012).

#### 3.2.1. Cytotoxicity Test

The in vitro cytotoxicity of the developed non-woven fabrics was tested by the direct and indirect contact with the monolayer cell culture L929 fibroblasts (ATCC CCL1). The mouse fibroblast-like cell line was maintained in Eagle’s medium at 2 × 10^6^ cells/mL. The culture medium with cells was seeded in Costar 24-well plates. Then, the cells were incubated over 24 h at 37 °C in 5% CO_2_ in air. In the indirect method, the monolayer cell culture was covered by 1000 µL of nonpolar and polar extracts from the biomaterials. The polar extract was prepared using Eagle’s medium without calf serum, supplemented with 100 U/mL penicillin, 100 μg/mL streptomycin, and 2 mM/mL l-glutamine. The non-polar extract is further characterized by the participation of a 2% calf serum in relation to the composition of the polar extract. In the direct method, a minimum 10% of the surface of the cell culture area was covered by the biomaterial [50]. Then, the cells were incubated at 37 °C in 5% CO_2_ in air for 24 h, 48 h and 72 h. Cell growth, morphology and viability (Trypan Blue Staining) were determined using image analysis methods. As the control, the same material but without the IGF1-containing microspheres was used. The level of toxicity was defined according to the requirements presented in EN ISO 10993-5:2009 [51].

#### 3.2.2. Genotoxicity Test

The investigations of the genotoxicity of non-woven implants was conducted with an Ames test (Xenometrix, Endotell GmBH, Allschwil, Switzerland) with four strains of *Salmonella typhimurium*, TA 98, TA 100, TA 1535, TA 1537, and two strains of *Escherichia coli* wp2 (uvrA and (pKM101)) with or without the metabolic activation of the rat liver S9 microsomal fraction [34]. The bacterial strains have introduced point mutations and are unable to synthesis the amino acids histidine (*E. coli*) or tryptophan (*S. typhimurium*). Growth in an environment that is reasonably free of these amino acids becomes possible after the reversion mutations by a factor mutagen. The mutagenic potential is evaluated by exposures to the bacterial strains with the prepared different solution of extracts of the test materials and the identification of a possible reversion. 

The experimental materials were extracted in a physiological NaCl solution in a proportion of 3 cm^2^ total surface area of a material over 1 mL of liquid by shaking (250 rpm) for 72 h at 37 °C. The test was conducted using well plates. Bacteria are pre-exposed to extracts of biomaterials as well as negative and positive controls with substrates containing the minimum quantity of histidine and tryptophan for two cell divisions. Next, cultures are diluted in conditions without histidine and tryptophan. The metabolism of the growing colonies changes the pH and composition of the substrate, causing a measurable colour reaction [34]. The test results are considered positive when the number of wells containing revertants is at least three times greater than that of the negative control. The test results were considered reliable when the average number of wells with a positive reaction (with revertants) in the negative control does not exceed the following values: 8 for *Salmonella typhimurium* strains TA 98, TA 1535, TA 1537 and 12 for strain *Salmonella typhimurium* TA 100 and *Escherichia coli* WP2 in the positive control of at least 25 for all strains *of Salmonella typhimurium* with and without metabolic activation *Escherichia coli* WP2 without activation of S9 fraction and three times the spontaneous reversion strain of *Escherichia coli* WP2 the activation of S9 fraction [34,51]. The statistical significance of differences in the number of revertants between experimental samples and negative controls were tested by a one-sided *t*-test. The differences were considered statistically significant at *p* ≤ 0.05. 

#### 3.2.3. Chronic Toxicity Studies after Implantation

Chronic toxicity was evaluated after the intra-osseous implantation of the tested materials for the period of the six months. The tests were performed on male and female rabbits of a New Zealand breed that had an average weight of 2.7 kg (±200 g). The rabbits were kept in the standard conditions of laboratory breeding, they were kept singly in cages under controlled humidity (28–37%) and temperature (16–20 °C). Animals had free access to water and were fed with a standard pelleted feed for rabbits (LSK, Fodder Plant, Motycz, Poland), with an average daily consumption of between 50 to 70 g per rabbit. The animals were divided into two experimental groups with PLGA + IGF1 or PLGA/PHB + IGF1 and one control group.

##### Surgical Procedures

Beginning 24 h before the scheduled surgical procedures, rabbits were subjected to fasting with access to water. An area of approximately 5 cm × 5 cm of the fur around the hip was removed mechanically. Rabbits were anesthetized with an intramuscular injection of anaesthetic mixture: xylazine at a dose of 5 mg/kg and ketamine (Biowet, Pulawy, Poland) a dose of 35 mg/kg. Full analgesia was obtained 10–15 min after the injection and lasted for 60–80 min. The full effect lasted for 120–140 min. After complete analgesia, at the height of the hips, the skin was disinfected with SkinSept Color (Ecolab, St. Paul, MN, USA) and incisions of 4–5 cm long were made, running along the base of the proximal femur. Then, the lesser and the greater trochanter were exposed, in which two holes having a diameter of, 3 mm and a length of 6 mm were drilled. In these cavities, the two test samples were placed on the right and two on the left side. The muscles and soft tissues were closed with a single surgeon’s knot of MonoPlus 3–0 (B Braun Medical Coe., Rubi, Spain) absorbable sutures. The skin was closed with a single stitch of Novosyn 2/0 (B Braun Medical Coe.). PLGA + IGF1 or PLGA/PHB + IGF1 were implanted into a femur trochanters of a rabbit, with four items in each animal for six months. Each investigated group was composed of eight animals (four males and four females). The animals in which there were no materials implanted were subjected to a surgical procedure identical to the animals in the tested groups, constituting the control. In the postoperative period, animals were housed in single cages with free access to water and food under constant medical-veterinary care. The overall health of the rabbit was evaluated, with special emphasis on the healing of surgical wounds, active and passive mobility of the hip and food intake. During the whole observation period, the condition, weight gain of the animals, feed consumption were monitored. Skin and natural orifices of the body of the animals were observed on the macroscopic scale and the potential changes in the muscles and the skeletal system were evaluated [35,52,53,54].

##### Blood Test

The blood for the tests was taken from the middle artery of the rabbits’ ear, the tested group and control, before surgery—time 0 and 90 and 180 days after surgery.

• Hematological Tests

The haematocrit value (HCT), haemoglobin concentration (HGB), red cells (RBC), platelet cells (PLT) and white cells (WBC) were counted with regard to leukogram: percentage of granulocytes, lymphocytes and other cells—monocytes, basophilic and eosinophilic granulocytes (MID) were determined in the full blood. The tests were performed on a PE-6800 haematology for veterinary science instrument PE-6800 VET (Procan Electronic Inc., Shenzhen, China) [55].

• Biochemical Tests

In the serum, the protein (PROT), glucose (GLU), cholesterol (HDL), bilirubin (TBIL), creatinine (CREA), urea concentration (URE) and amylase (AMYL), alkali phosphatase (ALP), aminotransferase alanine (ALT) and aspartate (AST) were determined. The tests were performed on an analyser activity an Epoll 20 Bio photometer (Poll LTD. Sp. z o.o, Wraszawa, Poland) [56]. In the serum IGF1 (insulin-like growth factor 1) was determined, performed on a LIAISON activity analyser, by DiaSorin (Saluggia, Italy) [57].

• Coagulation System Test

Activated partial thromboplastin time (APTT), prothrombin time (PT), thrombin time (TT), and fibrinogen concentration (Fb) were determined in the blood plasma. The tests were performed on a coagulometer (Coag Crom 3003 by Bio-Ksel Sp. z o.o, Grudziądz, Poland) [58,59].

• Statistical Analysis

The studied results were subjected to statistical analysis with use of programme Statistica 9.0 (Stat Soft Polska sp. zoo, Kraków, Poland). Arithmetical mean and standard deviation were counted. Statistical essentiality of the studied groups in comparison with the control was estimated with test T for independent samples. It is assumed that the differences are statistically significant at * *p* < 0.05, ** *p* < 0.01.

##### Macroscopic Post-MortemExaminations 

At the planned study time point of six months after implantation, euthanasia was performed on rabbits by an intravenous injection of pentobarbital (trade name: Morbital, producer: Biowet, Pulawy, Poland) at doses of up to 80 mg/kg, administered in fractionated doses to achieve respiratory arrest and cessation of heart function. Prior to pentobarbital administration, the general health status of the animals was assessed. During the autopsy, the postoperative scar and place of implantation of the samples as well as the appearance of the internal organs of the peritoneal cavity and chest were evaluated. The important organs located outside the peritoneal cavity and chest were also evaluated, anatomical location, size, colour were assessed.

##### Histological Evaluation

Selected internal organs were subjected to histological evaluation of potential changes compared to the control. The following histological specimens were taken: liver, stomach, small intestine, large intestine, kidney, testes, uterus, heart and lungs. The sections of organs were fixed for 48 h in a 10% aqueous solution of formic formaldehyde in phosphate buffer. The samples were dehydrated in acetone (56 °C), overexposed in xylene at room temperature and embedded in paraffin blocks. Microtome (Leica Microsystems Inc., Bannockburn, IL, USA) sections were cut to approximately 4 μm. The prepared formulations were dyed with haematoxylin and eosin (HE) and by Van Gieson (VG) reagent and then sealed in a medium. The histological specimens were evaluated under an optical microscope (Olympus BX43, Olympus, Tokyo, Japan). Characteristic histological findings were documented photographically using computer software for analysis and image acquisition, cellSens (Olympus).

## 4. Results

### 4.1. Cytotoxicity Test

The conditions of the cultures contacted with tested materials (direct method) or with extracts from the materials (indirect methods) were assessed using image analysis.

**Figure 2:**
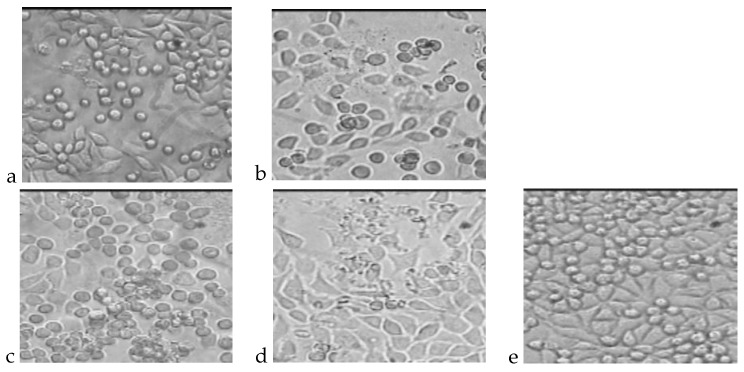
L929 cell cultures obtained during the cytotoxicity test of the direct method after 72 h of incubation. (**a**) Cells incubated with PLGA; (**b**) Cells incubated with PLGA + IGF1; (**c**) Cells incubated with PLGA/PHB; (**d**) Cells incubated with PLGA/PHB + IGF1; (**e**) L-929 culture without contact with tested materials.

The photos presented in Figure 2 show cultures of L-929 fibroblasts cells after direct 72 h of incubation. The total number of cellsand the percentages of living and dead cells are presented in Table 6.

The method of direct contact of the cells with material does not show the effect of substances, which can be released from the material during the extraction process. Thus, the assessment by the indirect method, as a comparison, was conducted.

**Figure 3:**
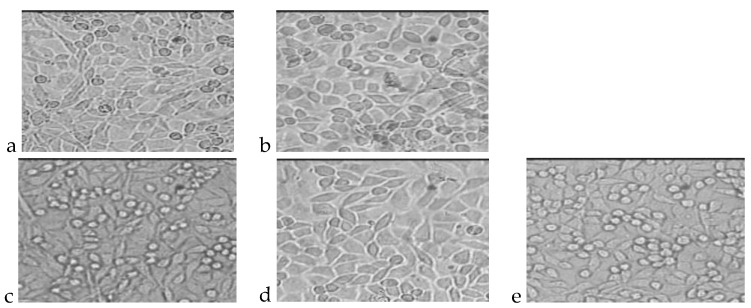
L929 cell cultures obtained during the cytotoxicity test of the indirect method (nonpolar extract) after 72 h of incubation. (**a**) Cells incubated with PLGA extract; (**b**) Cells incubated with PLGA + IGF1 extract; (**c**) Cells incubated with PLGA/PHB extract; (**d**) Cells incubated with PLGA/PHB + IGF1 extract; (**e**) L-929 culture without contact with extract from tested materials.

**Figure 4:**
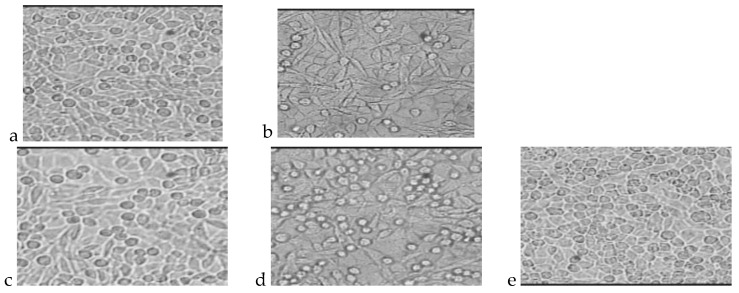
L929 cell cultures obtained during the cytotoxicity test of the indirect method (polar extract) after 72 h of incubation. (**a**) Cells incubated with PLGA extract; (**b**) Cells incubated with PLGA + IGF1 extract; (**c**) Cells incubated with PLGA/PHB extract; (**d**) Cells incubated with PLGA/PHB + IGF1 extract; (**e**) L-929 culture without contact with extract from tested materials.

The photos presented in Figure 3 and Figure 4 show cultures of L-929 cells after 72 h of incubation with polar or nonpolar extracts. The total number of cells and percentages of living and dead cells are presented in Table 7 and Table 8.

Tested materials: PLGA, PLGA + IGF1, PLGA/PHB and PLGA/PHB + IGF1 do not reveal any of the toxic effects for cell line L929—both in the studies performed by direct contact as well as the polar and non-polar extracts used under in vitro conditions.

### 4.2. Genotoxicity Test

The investigation of non-woven systems showed no genotoxicity against strains of *Salmonella typhimurium* TA 98, TA 100, TA 1535, TA 1537 and *Escherichia wp2 coli* with or without metabolic activation in rat liver S9 microsomal fraction (Ames test). The results are given in Table 9 and Table 10.

### 4.3. Chronic toxicity Studies after Implantation

#### Blood Parameters

**Figure 5:**
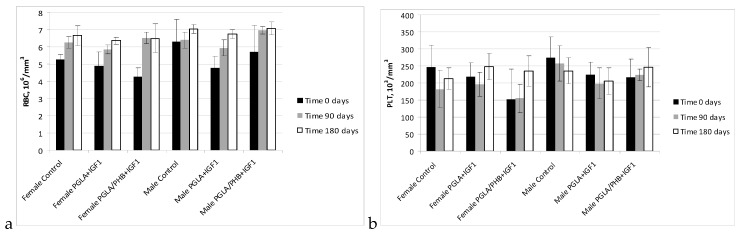
Red cell count (RBC) (**a**) and platelet cell count (PLT) (**b**) in blood of rabbits.

**Figure 6:**
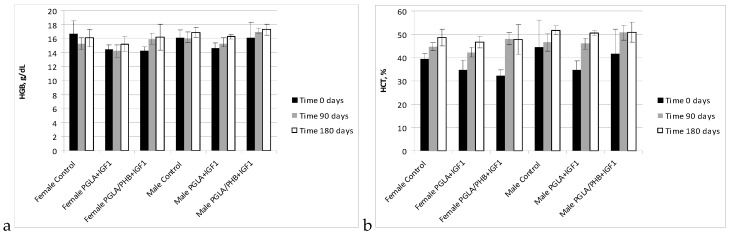
Haemoglobin concentration (HGB) (**a**) and value of haematocrit (HCT) (**b**) in blood of rabbits.

**Figure 7:**
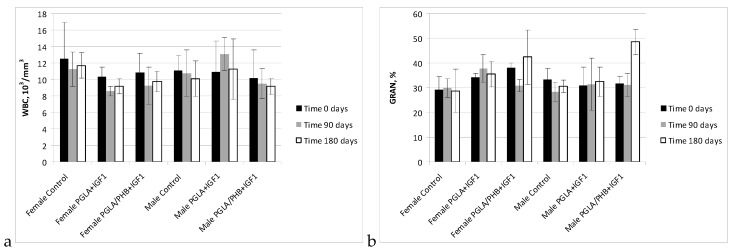
White cell count (WBC) (**a**) and percentage value of granulocytes (GRA) (**b**) in blood of rabbits.

**Figure 8:**
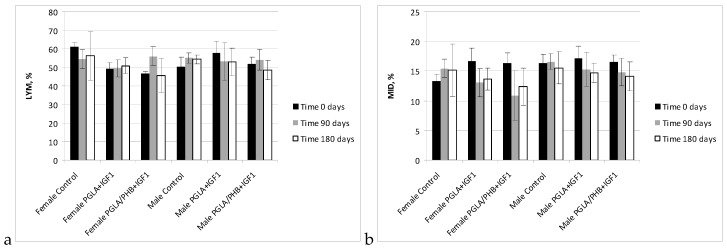
Percentage value of lymphocytes (LYM) (**a**) and other cells (MID) (**b**) in blood of rabbits.

The haematology blood of rabbits (male and female) with implanted samples of bone implants made from PLGA copolymer and its mixture with 10% of PHB (PLGA/PHB), with both materials containing insulin-like grow factor IGF1, has shown that the materials do not cause significant changes in the values of red, white blood cell and platelet parameter. The values of these parameters were comparable to the control group. The average values ± standard deviations are given in the form of Figure 5, Figure 6, Figure 7 and Figure 8. Haematological tests of rabbit blood (females and males) with implanted specimens of PLGA + PHB and PLGA implants showed that they did not cause significant changes in the parameters of haematological parameters. The values of these parameters were comparable to the control group values.

**Figure 9:**
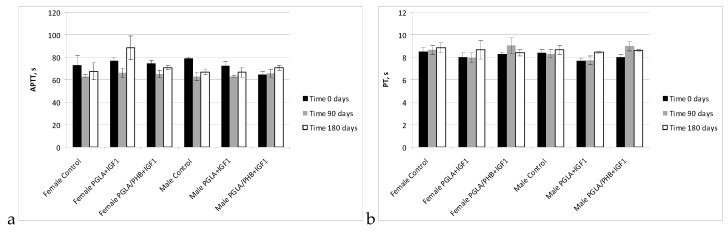
Activated partial thromboplastin time (APTT) (**a**) and prothrombin time (PT) (**b**) of blood of rabbits.

**Figure 10:**
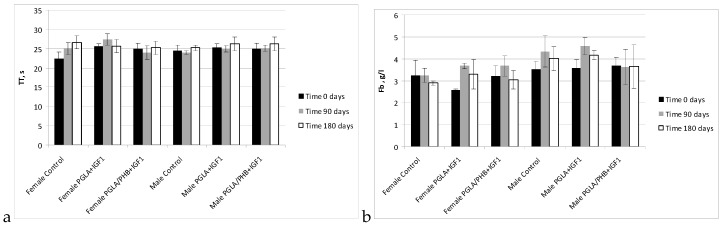
Thrombin time (TT) (**a**) and concentration fibrinogen (Fb) (**b**) of blood of rabbits.

In studies of the coagulation time of activated partial thromboplastin time, prothrombin time, thrombin time, and fibrinogen time in the treated groups (males and females) for PLGA + IGF1 and PLGA/PHB + IGF1 were in the range of the control group. The average values ± standard deviations are given in the form of Figure 9 and Figure 10. In the coagulation tests, partial thromboplastin time after activation, prothrombin time and thrombin time and fibrinogen concentration for the test groups (females and males) in PLGA and PLGA + PHB were within the range of the control group.

**Figure 11:**
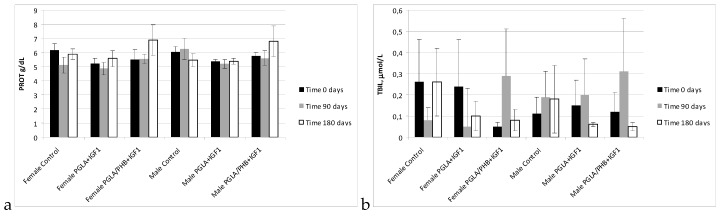
Protein (PROT) (**a**) and total bilirubin (TBIL) (**b**) concentration in blood of rabbits.

**Figure 12:**
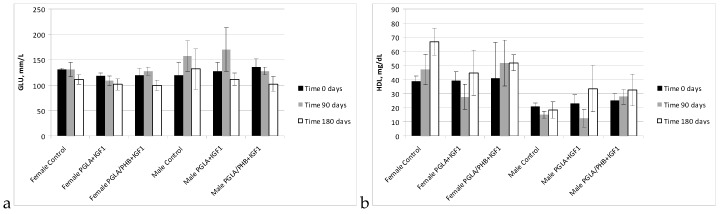
Glucose (GLU) (**a**) and cholesterol (HDL) (**b**) concentration in blood of rabbits.

**Figure 13:**
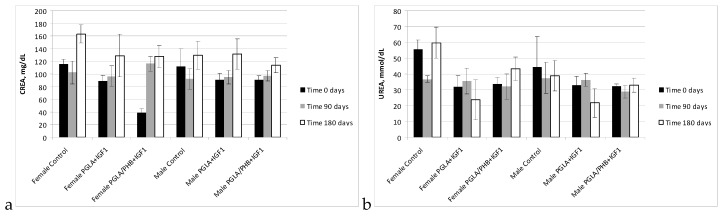
Creatinine (CREA) (**a**) and urea (Urea) (**b**) concentration in blood of rabbits.

**Figure 14:**
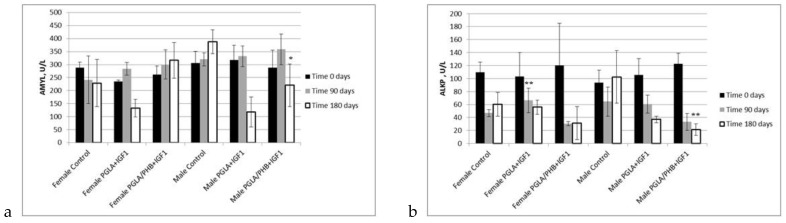
Amylase (AMYL) concentration (**a**) and alkali phosphatase (ALKP) activate (**b**) in blood of rabbits. * statistically significant differences at *p* < 0.05, ** statistically significant differences at *p* < 0.01.

**Figure 15:**
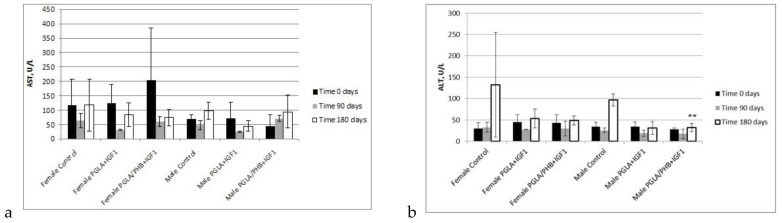
Aminotransferase aspartate (AST) (**a**) and aminotransferase alanine (ALT) activate (**b**) in blood of rabbits. ** statistically significant differences at *p* < 0.01.

During the biochemical tests of the blood of animals with PLGA + IGF1 and PLGA/PHB + IGF1 implants, no significant differences in the values of the relevant amylase, aspartate aminotransferase, alanine, γ-glutamyl transpeptidase, alkaline phosphatase, CK, and bilirubin, total protein, glucose, cholesterol, triglycerides, urea, creatinine, or insulin-like growth factor were observed between the test group and the control group—females and males. The observed changes in the values of the parameters recorded do not exceed the range of 20–30% of the reference values, which would have diagnostic significance. These changes are mainly observed after 90 days of implantation of samples made from PLGA + IGF1 and PLGA/PHB + IGF1 and can attest to the process of resorption of the implant. After 180 days, the designated parameters are similar to those in the control group. Results are presented in the form of graphs at the Figure 11, Figure 12, Figure 13, Figure 14 and Figure 15.

Results of the haematological, coagulation and biochemical tests of blood of rabbits performed 90 days and 180 days after implantation in the trochanter of femur of ossicular implants (PLGA + PHB and PLGA), presented in graphs, showed that the evaluated materials in the tested animal groups (females, males) generally, do not affect significantly the change in the value of these parameters compared to the value of control groups. The only observed, statistically important changes, were noticed for assessment of amylase (AMYL), alkali phosphatase (ALKP) and aminotransferase alanine (ALT) in male group after 180 days from implantation of samples made from PGLA/PHB + IGF1. The differences were compared to the value of control group. Moreover, in the case of assessment of concentration of alkali phosphatase, some changes, with *p* < 0.01 were also observed for female group after 90 days from implantation of PGLA + IGF1. These changes can attest to the process of resorption of the implant.

The measured values are comparable with the control values and fall within their reference values. The observed changes in the values of parameters indicate the ongoing biodegradation and resorption process in the tissue. Blood tests have diagnostic implications because their values allow us to evaluate the functioning of internal organs.

**Figure 16:**
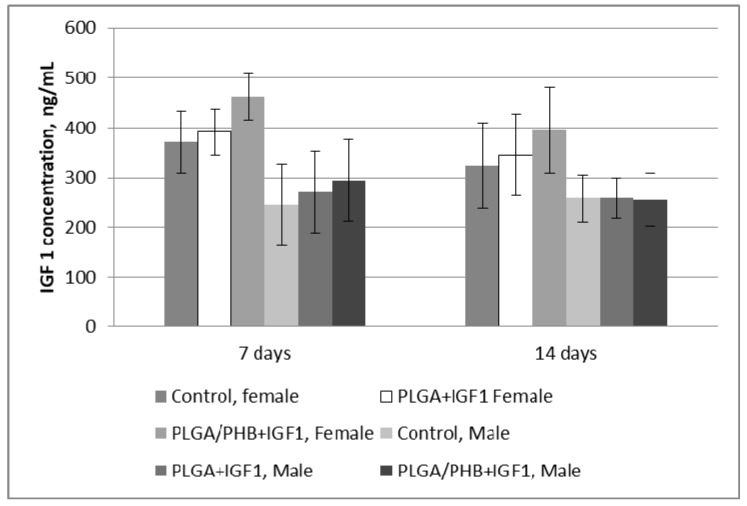
Insulin-like growth factor 1 (IGF1) concentration of in blood after 7 and 14 days from implantation.

Testing of blood parameters is of diagnostic importance, as their values allow for the evaluation of the functioning of internal organs. Presented results of the research indicate the proper function of internal organs. Additionally, the concentration of IGF in blood of female and male rabbits was assessed. Changes of the IGF1 concentration after 7 and 14 days from implantation are given at the Figure 16.

Obtained results shows, that the IGF1 concentration in rabbit blood, both female and male do not change after 7 and 14 days, and observed differences are statistically non-important. The activity of IGF 1 will be confirmed during in vivo tests—local effect after implantation.

### 4.4. Macroscopic and Histological Research

In the postoperative examinations of the animals, both for the examination and control groups, the subjects did not show any changes in the skin, bone or muscle. The natural orifices (outer ear, eyes, nostrils, mouth, and anus) and external genital organs showed no lesions. No differences in feed consumption, weight gain or overall condition of animals between the control and tested groups were observed during the 6th month period. The postoperative wounds, after 6 months, completely healed by first intention were macroscopically invisible.

In the autopsy examinations, in the peritoneum and chest, all the organs were arranged properly, with preserved anatomical shape, colour and size. No pathological changes were observed macroscopically in any of the treated organs. 

**Figure 17:**
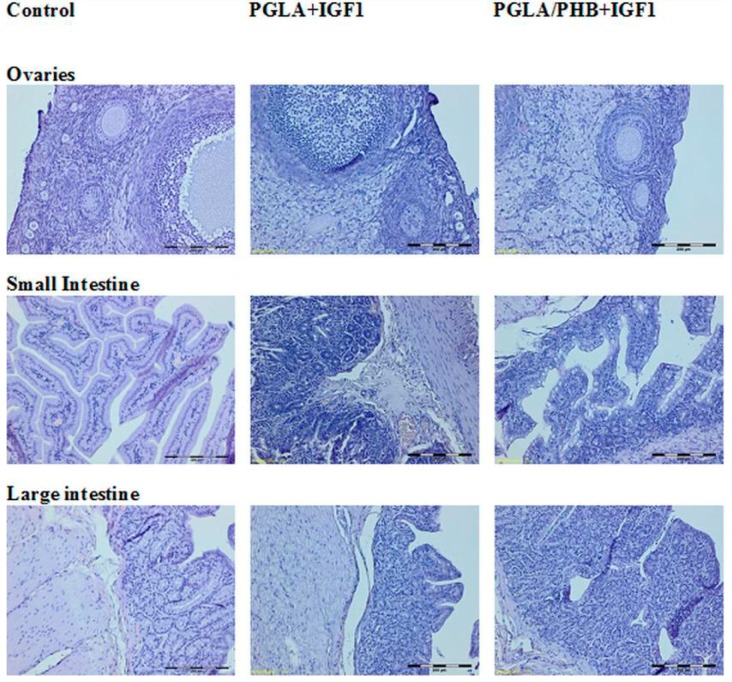
Microscopic images of internal organs (Dye HE. MG 100×).

**Figure 18:**
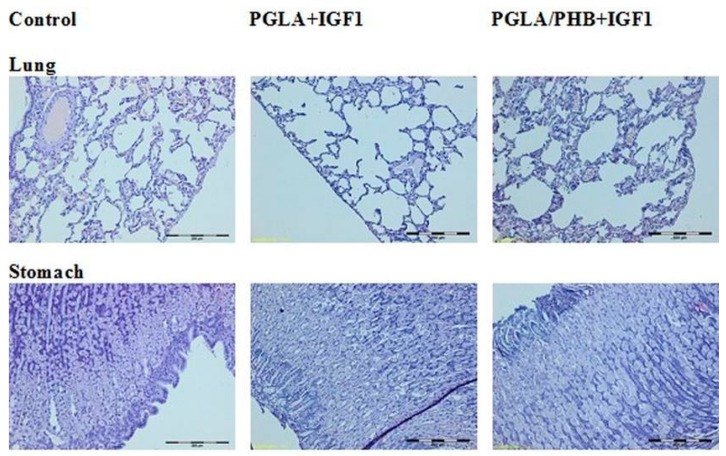

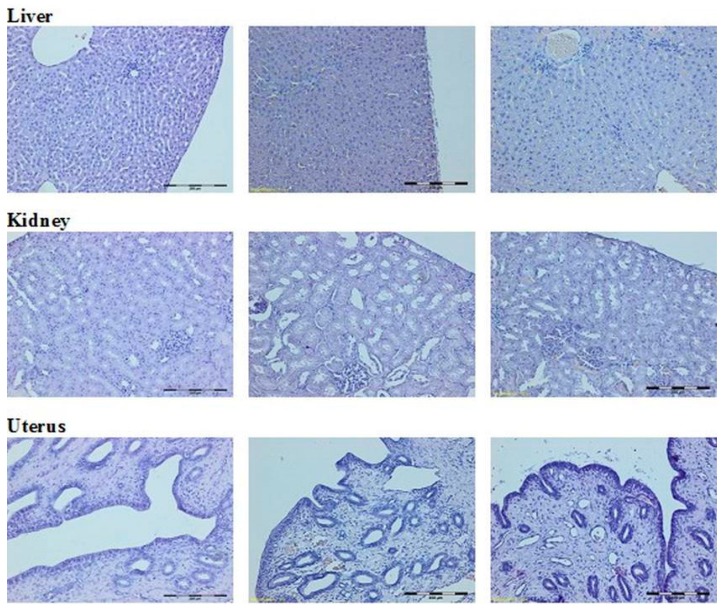
Microscopic images of internal organs. (Dye HE. MG100×).

**Figure 19:**
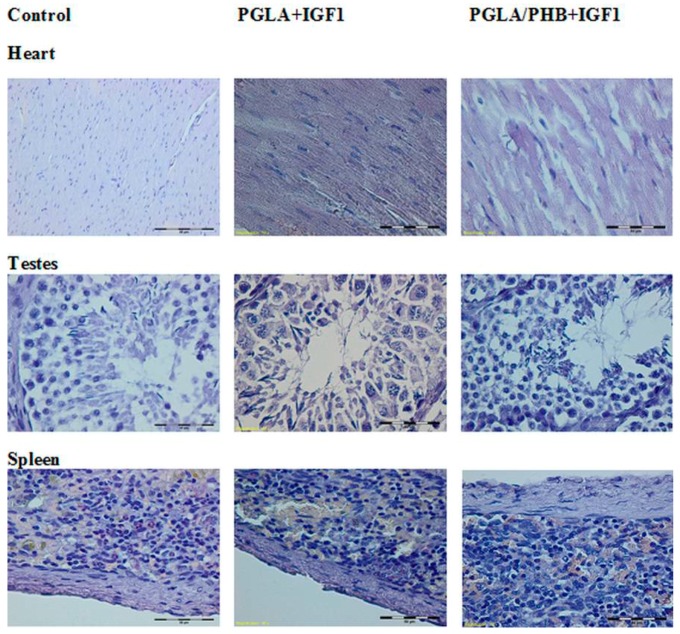
Microscopic images of internal organs. (Dye HE. MG 400×).

The histological study of microscopic images of kidney, lung, heart, liver, small and large intestine, spleen and ovaries, uterus and testes of animals from experimental groups showed no differences compared to the control groups. The internal organs in both groups showed no pathological changes (Figure 17, Figure 18 and Figure 19).

## 5. Discussion

A new technology for producing porous, multilayer, fibre-based bone implants from PLGA and PHB was developed. To obtain increased osteostimulation, implants were supplemented with nanoparticles with HAp and microspheres containing IGF1. The assumption made when using such materials was that the nanoparticles containing HAp should accelerate the process of bone remineralisation, whereas the presence of IGF1 should influence the process of cell proliferation and cause faster rebuilding of the destroyed tissue. The presented multi-layered construction of the material consisted of two different types of nonwoven materials to ensure better behaviour in the tissue regeneration. A needle-punched layer, built by open pores with a diameter of approximately 150 μm contains characteristics typical for osteoinductive structures, where cells can easily grow inside the structure [2]. At the same time, the nanofibrous layer creates a good surface for cell immobilization, as well as, thanks to the incorporation of the HAp, better activation of the remineralisation process. This layer is also a barrier for microspheres incorporated into the structure of the material. In the future, the nanoporous layer can be replaced by another type of material, like a microporous membrane or foam. The application of the IGF1 peptide caused the process of sterilisation of the material to be realized in two steps to avoid the risk of peptide denaturation.

Biological studies included the evaluation of the cytotoxic in vitro activity, which allowed for a preliminary evaluation of all the produced bone implant materials. Afterward, in vitro genotoxicity and chronic toxicity in New Zealand rabbits were subjected to a final bone implant prototypes supplied with IGF1, i.e., PLGA + IGF1 and PLGA/PHB + IGF1. The in vitro cytotoxic effect on the L929 cell line, both in the method of direct and indirect contact, showed no cytotoxic effect of the investigated implants PLGA + IGF1 and PLGA/PHB + IGF1 or the control materials PLGA and PLGA/PHB.

The in vitro genotoxicity studies with the experimental materials did not show mutagenic activity against bacteria used in the Ames test with and without metabolic activation. Thus, they do not cause a frameshift mutation or base pair substitution. The mutagenicity observed in the cultures from being in contact with both tested materials was at the level of spontaneous reversion and was significantly lower than the cells with the positive control.

Other authors using similar assays in the study of the mutagenicity of biomedical products and medicaments where PGLA were used as vehicle carriers also did not show the effect of these polymers on the end result [60]. Although tin compounds exhibited cytotoxic activity against the cells, mutagenic studies on metallic tin and its compounds have been negative. Long-term animal carcinogenic studies have shown fewer malignant tumours in animals exposed to tin than in controls [13]. The complexity of potential mutagenic and carcinogenic activity is demonstrated by the study of engineered nanomaterials used in wide range of commercial products and biomedical applications based on the nanosilver and magnetite nanoparticles. Two types of nanosilver, spherical nanoparticles and fibrous nanorods/wires, and magnetite nanoparticles differing in surface modifications among others with poly(lactide-*co*-glycolic acid) were included in this study. The results revealed that fibrous shape underlies the mutagenic and carcinogenic potential of nanosilver while surface chemistry affects the biosafety of magnetite nanoparticles [61].

The evaluation of systemic toxicity after intraosseous implantation PLGA/PHB + IGF1 or PLGA + IGF1 included clinical examinations of the animals, blood diagnostic tests, post-mortem examinations, and macroscopic and microscopic examinations of selected internal organs.

Diagnostic evaluation parameters included blood haematology, biochemistry, and coagulation for males and females in the control group and experimental groups with the bone implants PLGA/PHB + IGF1 and PLGA + IGF1. The implants tested in groups of animals do not substantially affect the value of these parameters compared to those of the control groups [36]. The obtained results for the experimental groups are comparable to those of the control group and are within the scope of their reference values. The observed slight changes in values indicate the on-going process of resorption of implants’ fibres in the tissue. The tests of the IGF1 concentration in blood plasma after the implantation of materials containing microspheres with this peptide do not show important changes to the amount of IGF1 in the blood compare to the control. The concentration of IGF1 in the blood of female and male rabbits was comparable in both groups of animals. Tests of blood parameters have diagnostic significance because they allow assessment of the functioning of the internal organs.

We performed tests of biochemical blood parameters, characterizing the body’s general condition and function of internal organs: total protein—general, bone, renal, metabolic profile; glucose—a general, diabetic, pancreatic, metabolic profile; cholesterol—lipid profile, metabolic profile; bilirubin—a general, metabolic, creatinine profile; urea—general profile, renal, metabolic profile; amylase—pancreatic, liver profile; alkaline phosphatase—general, hepatic, cardiac profile; aspartate aminotransferase and alanine—general, hepatic, cardiac, metabolic profile; creatine kinase—muscular, cardiac profile. The results of the diagnostic tests indicate the proper function of the internal organs.

The autopsy of the animals, for both the control group and experimental groups did not show any skin, bone or muscle changes. The natural orifices (outer ear, eyes, nostrils, mouth, and anus) and external genital organs showed no lesions. In the peritoneum and chest, all the organs were arranged properly, with preserved anatomical shape, colour and size. None of the inspected treated organs sowed macroscopic lesions in the macroscopic or microscopic studies of internal organs: kidneys, lungs, heart, liver, small intestine, colon, and uterus, ovaries and testes of animals from experimental groups—females and males showed no difference from those of the control group. The internal organs in both groups showed no signs of a pathological condition.

Similar results were obtained in the evaluation studies of the effects of poly(lactide-*co*-glycolide) (PLGA) nanoparticles in vitro and in vivo compared to industrial nanoparticles including zinc oxide, ferrous oxide, and fumed silica. It was concluded that the toxic effects observed with various industrial nanoparticles was not observed with particles made of PLGA. The biodistribution of these particles warranted surface modification to avoid particle accumulation in the liver [62].

## 6. Conclusions

The presented results allow us to conclude that implants for bone tissue regeneration produced from the newly developed, zirconium-based copolymer of lactide and glycolide with insulin-like growth factor (PLGA + IGF1) as well as the implants produced from the blend of this copolymer with poly([*R*,*S*]-3-hydroxybutyrate) (PLGA/PHB + IGF1) do not show any negative influence on living organisms. The tested implants do not change in vitro the cell growth, morphology and viability in either direct or indirect test methods. The cytotoxicity level in vitro, was independent of the presence of an insulin-like growth factor IGF1. The Ames test conducted for implants from PLGA + IGF1 and PLGA/PHB + IGF1 does not show any mutagenic effect. In the chronic toxicity studies, the tested implants PLGA + IGF1 or PLGA/PHB + IGF1, do not cause essential changes in the values of blood parameters of animals subjected to the implantation. The values of the haematological, coagulation system and biochemical parameters are comparable to the control one and are in the range of the values before the surgery. The concentration of IGF1 in the blood plasma of rabbits was comparable in both tested and control groups of animals and the results were independent of sex. The obtained results of blood parameters macroscopic and histological evaluation indicated the correct functions of the internal organs after implantation. Based on the research, it was found that the implants made from PLGA/PHB + IGF1 and PLGA + IGF1 materials do not show chronic systemic toxicity. Tests conducted in vitro (cytotoxicity and genotoxicity) as well as tests conducted in vivo using rabbits show the proper biological reaction of living organism on the presented implants.

## Figures and Tables

**Table 1:** Characteristic of the fibres used for manufacturing of the implants.

No.	Polymer	Linear Mass, dtex	Tenacity, cN/tex	Elongation, %	Shrinkage, %	Crystallinity Degree, %
1	PLGA	6.02	24.1	40.4	5.0	14.0
2	PLGA/PHB	5.80	26.8	41.9	16.4	13.0

**Table 2:** Characteristics of the needle-punched non-woven fabrics used for macroporous layer of bone implants for osseous tissue regeneration.

Polymer	Surface Mass, g/m^2^	Thickness, mm	Apparent Density, kg/m^3^	Air Permeability, lm^−2^ s^−1^	Average Pore Diameter, nm	Total Pore Area, m^2^/g
PGLA	333.76	2.6	128.08	331	148,523.10	0.116
PGLA/PHB	318.13	2.2	144.55	409	158,719.30	0.085

**Table 3:** Characteristic of the nanoporous layer constituting the implant.

No.	Polymer	Surface Mass, g/m^2^	Thickness, mm	Apparent Density, kg/m^3^	Average Pore Diameter, nm	Total Pore Area, m^2^/g
1	PLGA	4.5	0.03	150.00	9.80	1.49
2	PLGA/PHB	20.4	0.07	219.43	16.90	0.56

**Table 4:** Implantable material characteristics.

No.	Polymer	Surface Mass, g/m^2^	Thickness, mm	Apparent Density, kg/m^3^	Air Permeability lm^−2^ s^−1^
1	PLGA	602.36	2.8	215.13	205
2	PLGA/PHB	613.24	2.8	219.01	202
3	PLGA + IGF1	665.95	2.8	237.84	198
4	PLGA/PHB + IGF1	692.34	2.7	256.42	178

**Table 5:** Characteristics of porous structure of implantable material.

No.	Polymer	Average Pore Diameter, nm	Total Pore Area, m^2^/g
1	PLGA	168,236.2	0.246
2	PLGA/PHB	171,522.9	0.205
3	PLGA + IGF1	172,877.1	0.139
4	PLGA/PHB + IGF1	181,042.3	0.133

**Table 6:** Number of cells and level of cytotoxicity after 72 h of incubation—direct method.

No.	Polymer	Dead Cells, %	Living Cells, %	Total Number of Cells, ×10^5^	Level of Cytotoxicity
1	PLGA–control	0	100	4.1	0
2	PLGA + IGF1	0	100	5.6	0
3	PLGA/PHB–control	1	99	3.8	0
4	PLGA/PHB + IGF1	0	100	4.7	0
5	L929 culture	0	100	6.8	0

**Table 7:** Number of cells and level of cytotoxicity after 72 h of incubation—indirect method (nonpolar extract).

No.	Polymer	Dead Cells, %	Living Cells, %	Total Number of Cells, ×10^5^	Level of Cytotoxicity
1	PLGA–control	0	100	5.2	0
2	PLGA + IGF1	0	100	4.5	0
3	PLGA/PHB–control	0	100	4.6	0
4	PLGA/PHB + IGF1	1	99	4.7	0
5	L929 culture	0	100	6.4	0

**Table 8:** Number of cells and level of cytotoxicity after 72 h of incubation—indirect method (polar extract).

No.	Polymer	Dead Cells, %	Living Cells, %	Total Number of Cells, ×10^5^	Level of Cytotoxicity
1	PLGA–control	0	100	7.9	0
2	PLGA + IGF1	2	98	5.9	0
3	PLGA/PHB–control	0	100	6.1	0
4	PLGA/PHB + IGF1	0	100	5.4	0
5	L929 culture	0	100	7.2	0

**Table 9:** Number of positive cells in Ames test of PLGA + IGF1.

Dose	TA 98		TA 100		TA 1535		TA 1537		*E. coli* wp2	
cm^2^/mL	−S9	+S9	−S9	+S9	−S9	+S9	−S9	+S9	−S9	+S9
Spontaneous reversion	0.56 ± 0.53	1.67 ± 2.08	0.33 ± 0.58	0.67 ± 0.58	0.33 ± 0.58	2.00 ± 1.73	1.67 ± 1.53	4.33 ± 2.31	6.67 ± 1.53	5.00 ± 2.65
0.09375	0.33 ± 0.58	0.67 ± 1.15	0.33 ± 0.58	2.33 ± 0.58	1.67 ± 1.15	1.70 ± 1.53	0.00 ± 0.00	3.00 ± 1.00	10.00 ± 4.58	5.00 ± 1.00
0.1875	1.67 ± 1.15	1.33 ± 0.58	0.00 ± 0.00	1.67 ± 1.53	0.67 ± 1.15	1.67 ± 0.58	1.33 ± 1.53	2.00 ± 1.00	8.33 ± 5.13	3.67 ± 5.51
0.375	0.33 ± 0.58	2.67 ± 2.31	0.33 ± 0.58	1.33 ± 0.58	0.00 ± 0.00	1.33 ± 0.58	1.00 ± 1.00	3.67 ± 0.58	8.33 ± 2.52	5.00 ± 0.00
0.75	0.67 ± 1.15	5.00 ± 1.00	0.00 ± 0.00	2.00 ± 1.00	1.00 ± 0.00	2.67 ± 0.58	0.67 ± 0.58	2.33 ± 1.53	8.00 ± 3.46	7.67 ± 8.02
1.5	0.00 ± 0.00	2.67 ± 3.06	1.00 ± 1.00	2.00 ± 1.00	0.67 ± 0.58	1.00 ± 1.00	0.67 ± 0.58	3.00 ± 0.00	9.67 ± 4.62	8.00 ± 7.00
3	2.00 ± 1.00	3.00 ± 0.00	1.67 ± 1.53	0.67 ± 0.58	0.33 ± 0.58	1.67 ± 1.15	0.67 ± 0.58	2.33 ± 0.58	6.67 ± 1.53	9.33 ± 3.51
Positive control	40.67 ± 2.08	47.67 ± 0.58	36.33 ± 1.53	46.33 ± 2.08	48.00 ± 0.00	27.67 ± 2.08	48.00 ± 0.00	32.67 ± 7.37	45.33 ± 1.15	43.00 ± 1.00

**Table 10:** Number of positive cells in Ames test of PLGA/PHB + IGF1.

Dose	TA 98		TA 100		TA 1535		TA 1537		*E. coli* wp2	
cm^2^/mL	−S9	+S9	−S9	+S9	−S9	+S9	−S9	+S9	−S9	+S9
Spontaneous reversion	0.56 ± 0.53	1.67 ± 2.08	0.33 ± 0.58	0.67 ± 0.58	0.33 ± 0.58	2.00 ± 1.73	1.67 ± 1.53	4.33 ± 2.31	6.67 ± 1.53	5.00 ± 2.65
0.09375	1.67 ± 1.53	1.33 ± 1.15	0.33 ± 0.58	1.33 ± 0.58	0.33 ± 0.58	1.67 ± 2.08	2.33 ± 2.52	2.00 ± 2.65	4.00 ± 3.61	6.67 ± 3.06
0.1875	0.33 ± 0.58	1.67 ± 0.58	0.67 ± 0.58	0.33 ± 0.58	0.67 ± 0.58	2.67 ± 2.08	1.33 ± 1.53	2.33 ± 1.53	6.33 ± 3.51	1.67 ± 1.15
0.375	1.67 ± 2.89	3.33 ± 0.58	0.67 ± 1.15	2.33 ± 2.52	1.00 ± 1.73	2.00 ± 1.00	1.33 ± 2.31	1.33 ± 1.53	13.00 ± 2.65	5.67 ± 4.73
0.75	1.00 ± 1.00	1.67 ± 1.53	0.33 ± 0.58	2.00 ± 3.46	0.33 ± 0.58	2.00 ± 1.00	1.00 ± 1.00	4.00 ± 3.00	9.67 ± 2.52	3.00 ± 1.73
1.5	1.00 ± 1.00	2.00 ± 1.73	0.33 ± 0.58	1.00 ± 0.00	0.67 ± 0.58	1.33 ± 1.53	2.00 ± 1.00	1.67 ± 0.58	8.33 ± 1.53	2.00 ± 2.00
3	1.67 ± 1.15	2.33 ± 2.52	0.67 ± 0.58	1.33 ± 1.53	0.33 ± 0.58	2.00 ± 1.00	1.67 ± 0.58	2.33 ± 0.58	6.00 ± 1.00	5.33 ± 1.53
Positive control	40.67 ± 2.08	47.67 ± 0.58	36.33 ± 1.53	46.33 ± 2.08	48.00 ± 0.00	27.67 ± 2.08	48.00 ± 0.00	32.67 ± 7.37	45.33 ± 1.15	43.00 ± 1.00

The conclusion from the presented results is that the materials do not show any genotoxic effect.

## Supplementary Materials

Supplementary File 1
