# Supplementary material for: Biological Properties of Low-Toxicity PLGA and PLGA/PHB Fibrous Nanocomposite Implants for Osseous Tissue Regeneration. Part I: Evaluation of Potential Biotoxicity
Source: Molecules. 2017 Nov 29;22(12):2092. doi: 10.3390/molecules22122092 (PMC6149750; doi:10.3390/molecules22122092)
Supplement: Supplementary File 1 [file molecules-22-02092-s001.zip › Answer for Rev. 1..docx]

Dear Reviewer,

Thank you very much for Yours efforts and analysis of the manuscript and very helpful comments. We made important changes in our text. Incorporation of new text is marked on blue colour. The answers to most of the comments and remarks we have tried to include in the text.

1. **The authors presented a lot of data, but they are not easy to read. I suggest to concentrate the data to some significant data for presentation.**

Some data were removed or presented in the form of graphs.

2. **The graphic presentation must be better for reading than the raw digital data table.**

The biggest tables, connected with the presentation of blood examination we present on the form of graphs.

**3. The animal welfare committee were not stated in the manuscript**

The data of welfare committee was added.

**4. The aims are not clear in this manuscript.**

The aim of the work was cleared.

5. **In the histological study , the surgical protocol were not clear described in the manuscript.**

**The surgical protocol-** required data were added.

**6. The manuscript described the muscles and the skeletal system were evaluated .But the specimen of liver, stomach, small intestine, large intestine, kidney, testes, uterus, heart and lungs were taken . This protocols are not normal and meaningful . I suggest the authors should clear the meaning and protocol of animal study.**

The correction and explanation was done in the text.
