# Supplementary material for: Biological Properties of Low-Toxicity PLGA and PLGA/PHB Fibrous Nanocomposite Implants for Osseous Tissue Regeneration. Part I: Evaluation of Potential Biotoxicity
Source: Molecules. 2017 Nov 29;22(12):2092. doi: 10.3390/molecules22122092 (PMC6149750; doi:10.3390/molecules22122092)
Supplement: Supplementary File 1 [file molecules-22-02092-s001.zip › Answer for Rev. 2..docx]

Dear Reviewer,

Thank you very much for Yours efforts and analysis of the manuscript and very helpful comments. We made important changes in our text. Incorporation of new text is marked on blue colour. The answers to most of the comments and remarks we have tried to include in the text.

1. **The chapter of "Chemical and physical characteristics of ..." is not clear whether it is the results obtained in this study or previousl report. If they are obtained in this study, please move to the section of "Results".**

The organisation of the manuscript was changed according to your suggestions.

2. **Please move the exprimental information in the section of "Results" to the section of "Materials and Methods"..**

The organisation of the manuscript was changed according to your suggestions.

**3. Tables are too many and it is hard to understand the data.**

Part of the data from the tables is presented in the form of graphs..

**4. The section of "Discussion" is not found.**

In fact, the chapter “Summary” it was the discussion. We changed the title

5. **Summary is too long.**

Summary was deleted

**6. Please discuss the resorption rate of the materials.**

Literature indicates that the degradation of PLA-based materials is influenced by the nature of the material itself, the temperature, and the surface to weight ratio. At 37 ^0^ C degradation occurs during 90-210 days. At temp 70^0^ C -60 days. The prepared tested PGLA-based materials degraded between 3 and 6 months. ISO standards recommendations indicate that the chronic toxicity observation time of the implanted material in each species should be 10% of life of the animal, taking into account laboratory conditions [1]. Hence 6 months observation time.

1. PN-EN ISO 10993-11: 2009 Biological evaluation of medical devices -- Part 11: Tests for systemic toxicity
