# Supplementary material for: Biological Properties of Low-Toxicity PLGA and PLGA/PHB Fibrous Nanocomposite Implants for Osseous Tissue Regeneration. Part I: Evaluation of Potential Biotoxicity
Source: Molecules. 2017 Nov 29;22(12):2092. doi: 10.3390/molecules22122092 (PMC6149750; doi:10.3390/molecules22122092)
Supplement: Supplementary File 1 [file molecules-22-02092-s001.zip › Answer for Rev. 3..docx]

Dear Reviewer,

Thank you very much for Yours efforts and analysis of the manuscript and very helpful comments. We made important changes in our text. Incorporation of new text is marked on blue colour. The answers to most of the comments and remarks we have tried to include in the text.

1. **According to the instructions for Authors, the manuscript should be divided in 5 sections (Introduction, Results, Discussion, Materials and Methods, Conclusions), the authors didn’t respect them. The introduction should define the purpose of the work and its significance and report the current state of the research field. In this paper, the introduction is too general and not well focus on the aim of the research; moreover the current state of the art is completed missing. Authors reported the obtained results through tables, in my opinion it is better summarize the results, by using graphs, in order to make easier the reading and comprehension of the manuscript.**

The organisation of the manuscript was changed according to your suggestions.
